# Immediate Infusion Reaction to Intravenous Risankizumab-rzaa but Subsequent Tolerance to Subcutaneous Risankizumab-rzaa

**DOI:** 10.14309/crj.0000000000001546

**Published:** 2024-11-06

**Authors:** David Choi, Benjamin McDonald, David T. Rubin

**Affiliations:** 1 University of Chicago Medicine Inflammatory Bowel Disease Center, Chicago, IL

**Keywords:** Skyrizi, risankizumab, IBD, Crohn's disease, inflammatory bowel disease

## Abstract

Risankizumab-rzaa (RZA) is a fully human immunoglobulin G monoclonal antibody (Mab) which has fewer immunogenic concerns compared with chimeric Mabs. The pivotal trials of RZA found no significant differences in infusion reactions between drug and placebo. This case illustrates successful transition to subcutaneous RZA following a serious hypersensitivity reaction to intravenous RZA.

## INTRODUCTION

Crohn's disease (CD), a type of inflammatory bowel disease (IBD), is characterized by gastrointestinal ulceration and transmural inflammation.^1^ Risankizumab-rzaa (RZA) was approved by the Food and Drug Administration on June 17, 2022, for the treatment of moderately to severely active CD dosed with 3 intravenous (IV) infusions for induction and subsequent subcutaneous (SC) injections in maintenance.^2^ Phase 3 trials have demonstrated the safety of IV RZA with 1%–2% reporting an infusion-related reaction, < 1% developing serious hypersensitivity reactions, and 0% with anaphylactic reactions.^3^ We present a case of a patient who developed an immediate hypersensitivity reaction to RZA infusion but who subsequently tolerated the SC on-body injector.

## CASE REPORT

The patient is a 56-year-old woman with a history of Crohn's disease who required total proctocolectomy with the creation of an end ileostomy and subsequent additional small bowel resections and bowel-sparing stricturoplasties. Prior therapies included infliximab (developed an erythematous eruption without anti-drug antibodies) and inadequate response to adalimumab, ustekinumab, and upadacitinib. She had prior documented drug allergies to ciprofloxacin and sulfonamides. RZA was prescribed due to worsening abdominal discomfort and a fecal calprotectin (FCP) 529 mcg/g. Although vedolizumab is approved for use in CD, optimal positioning is before tumor necrosis factor α-antagonist exposure, before the development of disease-related complications. As a result, this therapy was not viewed as an optimal option for this patient at the time.^4^ The patient has documented the correlation of FCP to assess the disease activity and confirmed the use as a biomarker. She received the first RZA infusion without incident. However, 30 minutes into the 1 hour second infusion, she developed nausea, throat tightness, and rigors. The infusion was discontinued, and she was treated successfully with diphenhydramine 50 mg IV and methylprednisolone 40 mg IV.

Because of a lack of additional therapies to treat her active CD, we decided to forego the remaining IV doses and continue with standard subcutaneous dosing, which was started as soon as possible (ie, 2 weeks after reaction). She administered the SC with premedications: famotidine, diphenhydramine, and acetaminophen and had no reaction. Importantly, she has responded to therapy. After 6 months which included 3 additional SC doses of RZA, her symptoms improved and repeat FCP is 61 mcg/g. She has not used premedication since the first SC dose.

## DISCUSSION

RZA is a fully human immunoglobulin G monoclonal antibody (Mab) which has fewer immunogenic concerns compared with chimeric Mabs.^2,3^ The pivotal trials of RZA found no significant differences in infusion reactions between drug and placebo.^3^ One patient developed a serious hypersensitivity reaction (rash and elevated liver enzymes), requiring hospitalization in the phase 3 ADVANCE induction trial. We and others have reported hypersensitivity with IV ustekinumab (which is only one dose), with subsequent tolerance of SC maintenance dosing.^5,6^ In our prior case with ustekinumab, we proposed that ethylenediaminetetraacetic acid disodium salt dihydrate present in the IV but not in the SC formulation may explain these observed differences.^4^ However, the excipients with RZA are identical between the IV and SC, so the cause of this patient's reaction remains unknown (Table 1).^7^ It is also important to note, at the time of this reaction, there were no commercially available tests to assess for anti-RZA antibodies. If commercially available tests become available, it may be helpful to ensure the patient did not develop anti-RZA antibodies.

**Table 1.** Excipients found in risankizumab intravenous and on-body injector

Risankizumab-rzaa Intravenous	Risankizumab-rzaa On-body injector
Acetic acid	Acetic acid
Polysorbate 20	Polysorbate 20
Sodium acetate	Sodium acetate
Water	Water
Trehalose	Trehalose

There are other types of infusion reactions observed that appear similar to an anaphylactic reaction but are of a different etiology. For example, vancomycin has been attributed to a flushing syndrome that is defined as an infusion reaction caused by the rapid infusion of vancomycin.^8^ Typically, this presents as erythematous rash, pruritus, and less frequently hypotension and angioedema. The cause of this flushing syndrome is due to the non-IgE-mediated release of histamine from mast cells and basophils that is related to the infusion rate of the medication. If this occurs, the effect can be avoided with slower infusion rates of vancomycin and does not require the discontinuation of vancomycin. It is unclear whether RZA may have a similar reaction with the release of histamine due to the infusion itself rather than the medication.

A systematic review evaluated infliximab-related infusion reactions.^9^ Infliximab is a chimeric Mab and is associated with well-recognized infusion-related reactions. However, the mechanism behind these reactions is not well understood. The authors identified 5 potential etiologies of infusion-related reactions to infliximab: (i) cytokine-release syndrome, (ii) true anaphylactic reaction (IgE mediated), (iii) IgG anaphylaxis, (iv) complement activation, and (v) degranulation of mast cells and basophils. It may be challenging to distinguish between these causes due to overlap in clinical manifestations. However, management would vary based off the etiology. Our patient appeared to have an immediate infusion reaction to RZA, given the timing of the infusion; however, her ability to tolerate the SC formulation may indicate the patient had an alternative etiology.

As additional IBD therapies are available as both IV and SC, it is important to develop a better understanding of infusion reactions to IBD therapies and when patients should discontinue therapy or consider switching to an alternative formulation. This case illustrates that a patient who has a serious reaction to IV doses of RZA may be safely and successfully treated subsequently with the SC dosing.

## DISCLOSURES

Author contributions: D. Choi, B. McDonald, and DT Rubin contributed to the design of the manuscript, wrote and revised the manuscript, and gave final approval and are accountable for the information presented. D. Choi is guarantor of the article.

Financial disclosure: D. Choi is on the speaker bureau for Janssen Pharmaceuticals and Eli Lilly and has served as a consultant to Bristol Myers Squibb, Boehringer Ingelheim, Eli Lilly, AbbVie, and Janssen Pharmaceuticals. DTR has received grant support from Takeda and has served as a consultant for AbbVie, Altrubio, Apex, Avalo Therapeutics, Bristol-Myers Squibb, Buhlmann Diagnostics Corp, Celgene, Connect BioPharma, Intouch Group, Iterative Health, Janssen Pharmaceuticals, Lilly, Pfizer, and Takeda. He serves on the Board of Trustees for the Crohn's & Colitis Foundation and is on the Board of Directors for Cornerstones Health.

Informed consent was obtained for this case report.

## References

[1] Feuerstein JD Cheifetz AS. Crohn disease: Epidemiology, diagnosis, and management. Mayo Clin Proc. 2017;92(7):1088–103. 28601423 10.1016/j.mayocp.2017.04.010

[2] Choi D Sheridan H Risankizumab-rzaa BS. A new therapeutic option for the treatment of Crohn's disease. Ann Pharmacother. 2023;57(5):579–84. 36214282 10.1177/10600280221130450

[3] D'Haens G Panaccione R Baert F . Risankizumab as induction therapy for Crohn's disease: Results from the phase 3 ADVANCE and MOTIVATE induction trials. Lancet. 2022;399(10340):2015–30. 35644154 10.1016/S0140-6736(22)00467-6

[4] Dulai PS Boland BS Singh S . Development and validation of a scoring system to predict outcomes of vedolizumab treatment in patients with Crohn's disease. Gastroenterology. 2018;155(3):687–95.e10. 29857091 10.1053/j.gastro.2018.05.039 PMC6419724

[5] Krugliak Cleveland N Masching A Rubin DT. Hypersensitivity to IV ustekinumab but tolerance to subcutaneous ustekinumab in a patient with Crohn's disease. ACG Case Rep J. 2020;7(8):e00449. 32904020 10.14309/crj.0000000000000449 PMC7449253

[6] Thomas PWA Ferwerda G West RL Hoentjen F. Immediate infusion reaction to intravenous ustekinumab in three Crohn's disease patients: A case report and review of the literature. J Crohns Colitis. 2021;15(1):162–4. 32588044 10.1093/ecco-jcc/jjaa115 PMC7805788

[7] Abbvie. SKYRIZI (risankizumab-rzaa) [package insert]. U.S. Food and Drug Administration website (https://www.accessdata.fda.gov/drugsatfda_docs/label/2024/761105s027,761262s008lbl.pdf) (2024). Accessed May 22, 2024.

[8] Martel TJ Jamil RT King KC. Vancomycin flushing syndrome. In: StatPearls [Internet]. StatPearls Publishing: Treasure Island, FL, 2024 (https://pubmed.ncbi.nlm.nih.gov/29494112/). 29494112

[9] Lichtenstein L Ron Y Kivity S . Infliximab-related infusion reactions: Systematic review. J Crohns Colitis. 2015;9(9):806–15. 26092578 10.1093/ecco-jcc/jjv096 PMC4558633

